# Controlling new knowledge: Genomic science, governance and the politics of bioinformatics

**DOI:** 10.1177/0306312716681210

**Published:** 2017-01-05

**Authors:** Brian Salter, Charlotte Salter

**Affiliations:** Department of Political Economy, King’s College London, London, UK; Norwich Medical School, University of East Anglia, Norwich, UK

**Keywords:** bioinformatics, genomics, governance, ideology, politics

## Abstract

The rise of bioinformatics is a direct response to the political difficulties faced by genomics in its quest to be a new biomedical innovation, and the value of bioinformatics lies in its role as the bridge between the promise of genomics and its realization in the form of health benefits. Western scientific elites are able to use their close relationship with the state to control and facilitate the emergence of new domains compatible with the existing distribution of epistemic power – all within the embrace of public trust. The incorporation of bioinformatics as the saviour of genomics had to be integrated with the operation of two key aspects of governance in this field: the definition and ownership of the new knowledge. This was achieved mainly by the development of common standards and by the promotion of the values of communality, open access and the public ownership of data to legitimize and maintain the governance power of publicly funded genomic science. Opposition from industry advocating the private ownership of knowledge has been largely neutered through the institutions supporting the science-state concordat. However, in order for translation into health benefits to occur and public trust to be assured, genomic and clinical data have to be integrated and knowledge ownership agreed upon across the separate and distinct governance territories of scientist, clinical medicine and society. Tensions abound as science seeks ways of maintaining its control of knowledge production through the negotiation of new forms of governance with the institutions and values of clinicians and patients.

## Introduction

Innovation in biomedicine is supported by an infrastructure of knowledge governance at national and transnational levels (Salter, 2009, 2011; Salter and Salter, 2010; Waldby and Salter, 2008). As each new area of biomedicine emerges, it may generate demands for change in the existing modes of knowledge governance: demands that reflect not only the particular qualities of the innovation but also the political interests of those sponsoring it. These demands may engage smoothly with the dominant systems of governance or they may produce tensions when they imply redistributions of political and economic power in the production of biomedical knowledge and in the ownership of the benefits produced. Either way, the control of the emergence of new knowledge through the maintenance and construction of governance determines how the politics of the new domain play out. For no field is this more true than for genomic science, with its impressive portfolio of biomedical promise, economic potential and political ambition.

With the launch of the Human Genome Project (HGP) in 1990, biology acquired its first ‘big science’ project, enabling it to compete effectively with physics and chemistry for status, public attention and state resources (see, for example, Cook-Deegan, 1994; Watson, 1990). Integral to that competitive drive has been the claim that genomic science will ‘provide researchers with powerful tools to understand the genetic factors in human disease, paving the way for new strategies for their diagnosis, treatment and prevention’ (National Institutes of Health (NIH), 2015). A brave new market of genomic medicine appeared to beckon. Early supporters such as President Bill Clinton heralded the project as ‘an epoch-making triumph of science and reason’ (*The New York Times*, 2000). The imminent health dividend for state and society seemed an irresistible justification for genomic science investment.

The delivery of this dividend has proved elusive, as the banks of bio-data have multiplied whilst the route to their translation into medical practice has become ever more complex. By the early 2000s, genomic science faced a credibility crisis. It is here that the rise of bioinformatics becomes important as a direct response to the political difficulties faced by genomics in its quest to be a new form of biomedical innovation. Bioinformatics offered the means of retaining the impetus of the science, the trust of state and society and the flow of public resources. As Dame Janet Thornton, Director of the European Bioinformatics Institute (EBI), emphasized, ‘it will be the biomedical informatics that will allow translations from knowledge and research into medical practice, delivered through the doctors… in the clinics, in the hospitals and ultimately for the GPs’ (House of Lords Science and Technology Committee, 2009: 20). The political value of bioinformatics is that it acts as the bridge between the promise of genomics and its realization in the form of health benefits. Without bioinformatics and its professed ability to address the exponentially rising tide of genomic data, it is doubtful whether the genomics project would have maintained its political momentum beyond the late 1990s; current high profile initiatives such as the UK’s Genomics England in 2014 ($524 million) and President Obama’s Precision Medicine Initiative in 2015 ($215 million) might not have been launched (GEN News, 2014; Genomics England, 2015; The White House Office of the Press Secretary, 2015). But if bioinformatics was to work as a solution that maintained and enhanced the power of genomic science, governance control had to be assured. The epistemic incorporation of bioinformatics as the saviour of genomics had to be integrated with the operation of two key aspects of governance in this field: the definition and ownership of the new knowledge. Only in this way would genomic science be able to guarantee that it reaped the benefits of the very substantial change in the production of knowledge on which it was embarked. How was this political ambition achieved?

In this article, we first describe the political problem facing genomics and the position of bioinformatics as a possible solution to that problem. To what extent did genomics become the victim of its own promissory vision, expanding the expectations of the health benefits to come whilst lacking the scientific capacity to deliver, thus generating the political need for a fresh form of legitimation of its social value? Second, what shape has the construction of the governance of bioinformatics taken, in attempts to reinforce and sustain its power, and how has this governance been legitimated? Third, to what extent has this exercise in scientific control been challenged, as the production of genomic knowledge has been extended beyond the territory of science to the domain of clinical medicine? In what ways and with what effect has this transition exposed science’s governance control to novel pressures from the values and interests of clinicians and patients?

We gathered data in two phases. In the first, internet desk-based scoping exercises of existing policies on bioinformatics were conducted through the analysis of policy documents of state and non-state governance actors in the UK, China and India, secondary ‘state of the science’ reports and expert overviews, industry trend reports, and science journalism. The results of this phase were summarized in project working papers and used as the platform for the development of a semi-structured interview schedule (Datta, 2014; Zhou, 2014). Thirty-two interviews were conducted with leading bioinformaticians, other elite scientists (particularly in the field of genomics) and policy makers spread across the three countries. The distribution of the interviews by country and primary role (scientist or policy maker) is shown in Table 1. Often a scientist would have a secondary role and also act in a policy-making capacity through formal membership of a state organization and, in some cases, would have strong industry links. The numerical effect of this overlap of roles within the interview sample is shown in Table 1. The interviews were recorded, transcribed, and analysed employing the conceptual framework developed in the following two sections. Initial categories derived from this framework were applied to the interview data and then refined and re-applied in the light of the iteration and ‘fit’ between framework and data. We presented a draft of this paper at a workshop of invited bioinformaticians in March 2015, and we revised it in the light of comments we received.

**Table 1.** Number of interviews by country, and number of roles by country.

	China	India	UK	Total
*Interviews by country*				
Science	8	8	10	26
Policy	2	1	3	6
Total	10	9	13	32
*Roles by country*				
Science	10	8	10	28
Policy	6	5	7	18
Industry	1	1	4	6
Total	17	14	21	52

## A promissory problem and bioinformatics solution

The research funding market of biomedical innovation is characterized by an intense competition between scientific disciplines for access to the finite resources of government and private agencies. With state and private funding agencies insisting that science funding be linked to the productivity of a scientific field and its economic, health and social benefits (e.g. Cooksey, 2006), actors have an incentive to promise that their fields can deliver such benefits faster and more efficiently than others. An internal power play of ‘promissory politics’ is generated, characterized by an inflationary spiral of ‘hype and hope’ as scientists outbid each other in attempts to achieve advantage in the research funding market (Brown, 2003; Morrison, 2012; Petersen and Krisjansen, 2015). The very nature of the spiral, and the inherent risk of loss of credibility in the face of non-delivery of promised benefits, necessitates the continuing creation of fresh legitimations of a discipline’s economic and social value. From the 1980s to the early 2000s, biology faced the legitimation problem that the volume, complexity and variety of bio-data production outstripped the discipline’s capacity to conceptualize, coordinate, analyse, and interpret it (Ouzounis and Valencia, 2003). The fear of being overwhelmed by the amount of data generated was palpable and public (Butler, 2001; Reichhardt, 1999), with official bodies such as the US National Institutes of Health Research recognising that ‘the computers, algorithms, and software, let alone the support infrastructure, are not keeping up with the exponentially rising tide of data in biomedical research’ (Working Group on Biomedical Computing, 1999).

The bigger the promise, the higher the expectations and the bigger the risk that the anticipated social benefits will not be delivered. In the ‘big science’ of genomics (the HGP became known as the ‘Manhattan Project’ of biology) the problem was particularly acute and visible (Galison and Hevly, 1992; Lenoir and Hays, 2000). By 1998, the HGP had spent nearly $2 billion with relatively little progress other than the sequences for the model organisms (Contreras, 2011: 70). And although the human genome was sequenced in 2003, the path to the promised health benefits remained obscure. The credibility of the science and the belief in its industrial potential were under severe political threat (Nightingale and Martin, 2004). In the UK, for example, a series of reports on genomics from the UK House of Lords Science and Technology Committee and Department of Health in the 2000s reiterated the difficulties faced by genomics and the ‘painfully slow’ translation of scientific research into ‘patient benefit’ (Department of Health, 2003; House of Lords Science and Technology Committee, 2001, 2009). In the US, the 1998 White House report *Bioinformatics in the 21^st^ century* noted that the need for a bioinformatics answer to the translation problem was ‘perhaps most evident in the field of genomics, where sequencing data and related datasets are growing at an exponential rate, far outstripping efforts to manage and analyze these data’ (The White House Office of Science and Technology Policy, 1998: 6).

Bioinformatics promised the solution to this problem through the imposition of order on the myriad uncertainties of the vast arrays of genomic data and, in so doing, provided a fresh legitimation and a new impetus to genomic science. Defined by the National Institutes of Health (NIH) in 2000 as the ‘Research, development, or application of computational tools and approaches for expanding the use of biological, medical, behavioral or health data, including those to acquire, store, organize, archive, analyze, or visualize such data’, the very rapid development of the field has, in the view of NIH, subsequently embraced what was then described as ‘computational biology’, which included ‘The development and application of data-analytical and theoretical methods, mathematical modeling and computational simulation techniques to the study of biological, behavioral, and social systems’ – adding what is seen to be a much more creative and dynamic dimension to its promissory curve (National Institutes of Health, Bioinformatics Definition Committee, 2000).

Behind the NIH’s confident public definitions of bioinformatics lies a problematic political history of epistemic emergence characterized by a continuing struggle between mathematics and computing science, on the one side, and biology on the other (Lewis and Bartlett, 2013; Ouzounis and Valencia, 2003). Integrating epistemic domains is a quintessentially political task because disciplines are constituted not only in terms of intellectual constructs and practices but also in terms of institutions with their particular interests and ambitions (Gieryn, 1983; Lemaine et al., 1976; Whitley, 1976). When the kind of power inherent in the ambition of the genomics project is at stake, the negotiation of a new identity is likely to be long and arduous. Initially, the importation of mathematical and computer science knowledge and skills into biology in the 1980s had been filtered through the existing power structures of biology, a process that rendered bioinformatics acceptable as a service function to the biological paradigm (Leonelli and Ankeny, 2012: 29–31; see also Garcia-Sancho, 2012, 2015). There appeared to be a natural convergence between the partners, described as a ‘natural marriage’, albeit one where one partner was manifestly dominant over the other (Chow-White and Garcia-Sancho, 2012: 14).

Driven by the imperatives of genomic science, its need to deliver for state and society sooner rather than later, and the sheer scale of the genomic data being generated, this marital harmony soon evolved into a more abrasive relationship, with mathematics and computing becoming ever more assertive. Practices devoted to the extraction of inferences from data *in silico* have become sufficiently sophisticated that ‘computational tools for data analysis are assigned a prominent role in facilitating the extraction of patterns from data, while experimental work is conceived as means to verify and explain those patterns’ (Leonelli, 2012a, 50). The consequence is that the creative power in the interdisciplinary relationship is moving to mathematics and computer science. As the power transfer takes effect, it challenges the ways in which science is organized and practiced through the forms of collaboration, division of labour and integrative strategies (of models, data, theories, and software) set up to deal with the fact of big data. As a result, Leonelli (2012b) claims, ‘Data-intensive methods are changing what counts as good science’ (p. 2). Similarly, Stevens (2011) notes how ‘bioinformatics entails a fundamental shift in practices of knowledge production: computers have allowed the reorganisation and revaluation of biological work’ to the extent that this generates ‘new ways of being “productive” in biology and that this “productivity” is tied not only to new kinds of work, but also to new kinds of knowledge in biology’ (p. 218). Such is the significance of the power transfer that the traditional paradigm of hypothesis-driven research is being replaced by what has been termed ‘discovery science’, where the database is established first and the explanations of the patterns they contain follow later (Chow-White and Garcia-Sancho, 2012: 146). In the workplace, the *in silico* ‘dry labs’ of electronic databases and computation are becoming as important as traditional *in vivo* ‘wet labs’ as the primary location of disciplinary activity (Biotechnology and Biological Sciences Research Council [BBSRC], 2012). In this space, the identity of bioinformatics is being forged and its political value established through the confidence it generates in the future of biology. It is ‘through computerization – through the principles and practice of bioinformatics – that genomics has become “industrialized” and “commercialized”’ and scientifically productive (Stevens, 2011: 240). And through the enhanced credibility this delivers, bioinformatics has rendered genomics more productive politically.

The reward for the epistemic solution bioinformatics offered to the impasse faced by genomics was visibility and resources. In the UK, in 2009 the Department of Health duly recognized that ‘[t]he expansion in EMBL-EBI [European Molecular Biology Laboratory-European Bioinformatics Institute] data management capacity is vital in underpinning the sustainable development of the substantial investments in genetic, genomic and systems biology made by the Research Councils’ (p. 18). Announcing a £32 million investment in bioinformatics in February 2014, the UK Minister for Science David Willetts emphasized its ‘huge priority for government’ and its ‘potential to drive research and development, increase productivity and innovation and ultimately transform lives’ (Medical Research Council, 2014). His statement built on the promise of the *Strategy for UK life sciences* to make the UK ‘a world leader in genomics and bioinformatics’ (Department of Business, Innovation and Skills, 2012: 41). In the US, Obama’s Precision Medicine Initiative promised ‘a bold new research effort to revolutionize how we improve health and treat disease’ by engaging ‘a million or more Americans to volunteer to contribute their health data to improve health outcomes, fuel the development of new treatments, and catalyze a new era of data-based and more precise medical treatment’ (The White House, Office of the Press Secretary, 2015). Thus was the formal political narrative established with bioinformatics at centre stage. How can we best analyse the politics that have driven the translation of scientific opportunity into political gain?

## Science, ideology and the state

It behoves the scientific community to maintain a close relationship with the state in order to be able to guide, if not determine, the agenda of the research funding market (Greenberg, 2001).Supporting the core concordat between science and state is an infrastructure of embedded institutions and values designed to maintain the relationship’s authority and legitimacy, promote continuing engagement between the two partners and facilitate the addition of new, mutually beneficial, scientific dimensions to the agreement (Jasanoff, 2004). Political exchange is continuous, with scientists lending their expertise and authority to the activities of the state’s policy advisory system, and the state facilitating and legitimizing science’s system of self-regulation and supporting ideology (Jasanoff, 1994). Underpinning the concordat, and an essential component in its operation, is public trust that the concordat’s goals, values, rules and outcomes are organized for wide benefit.

In his work on the scientific elite of the UK and the US, Mulkay (1976a) emphasizes the power of the elite, arguing that it ‘operates as a “buffer group” [between science and state], successfully resisting instrumental demands from outside and maintaining considerable freedom for members of the academic research community to pursue their own “scientifically defined” interests’ (p. 445). Central to the operation of this power is the ideology of science. Developed and refined in the 19^th^ century, this ideology presents the pursuit of truth as an ultimate value and the independent scientific community as the uniquely qualified occupational group to achieve it (Daniels, 1967). Within the ideology, and legitimating the right of science to fulfill this special social role, are an array of operating principles, propagated by scientists, including rationality, communality, universalism, disinterestedness, impartiality and organized skepticism (Merton, 1973; Mulkay, 1976b: 637–638). In his work on the development after World War I of the American ideology of national science, Tobey (1971) charts the deliberate sponsorship by science of ‘an image of the scientist as a particularly virtuous personality’. (pp. 178–179) – and, it might be added, therefore trustworthy. Similarly, in the UK, King has shown how in the 1930s British scientists promoted an ideology that explicitly linked the values of disinterestedness, impartiality, objectivity and so on with the nature of scientific knowledge production (King, 1968). Moral worth, epistemic identity and the right to be trusted were presented as indivisible.

The significance of ideologies can be measured by the extent to which they are politically useful. In the case of science, its ideology enables it to claim that scientists are uniquely equipped to understand the contribution of knowledge to the national interest, that the pursuit of knowledge should be publicly funded but not publicly governed, and that the values of science ensure that scientists will automatically govern themselves according to their established normative codes of disinterest and objectivity. In his US study, Greenberg (1969) illustrates the practical application of this ideological power to the science-state relationship:

But though reins and restrictions existed, and the principle of accountability (loathsome to the scientists) was never absent, the essential point was that, in practice, scientists wrote most of the rules for the use of federal money; scientists staffed the agencies that dispensed the money, and scientists from the university community advised these same staff scientists on the distribution of the money. (p. 330)

Equipped with a high-status occupational ideology utilizing what Mulkay (1976b) describes as many and varied ‘vocabularies of justification’, Western scientific elites are able to use their close relationship with the state to pursue their collective interests, maintain their territories and, importantly for this paper, facilitate the emergence of new domains compatible with the existing distribution of epistemic power – all within the embrace of public trust (p. 653).

However, as yet the same is not true of the scientific elites of emerging economies such as China and India. The scientific community in these countries is relatively new and still building its epistemic identity, institutions, status and relationships with the state. In China, for example, although a scientific elite is emerging it is inexperienced and lacks the characteristics such as self-regulation and promotion by merit normally associated with successful scientific communities (Cao and Suttmeier, 2001; Suttmeier and Cao, 1999). There is a long way to go before this scientific elite achieves the ideological self-confidence of its Western colleagues. Lacking the political muscle derived from the historic relationships with states enjoyed by Western scientific communities, the scientific elites of the emerging economies remain largely supporting players in the politics of global science, with their entry to the transnational scientific networks contingent upon their attractiveness as potential partners in collaborative research (Wagner and Leydesdorff, 2005; see also Georghiou, 1998; Luukkonen et al., 1992; Miquel and Okubo, 1994). In bioinformatics, Chinese and Indian scientists recognize the fact of Western dominance and typically see their development in this field as behind the global pace, describing themselves as ‘4-5 years behind the West’ [Interview 18, senior bioinformatics manager – China] and ‘we’re always laggards’ [Interview 14, bioinformatician – India]. A director of a Chinese genomics research centre commented: ‘Bioinformatics in China is still at a relatively early stage, with few internationally influential articles, databases, algorithms, and software. The collaboration between bioinformatics research and experimental biology is not adequate’ [Interview 37].

## Ideology, legitimation and the governance of innovation

Western science therefore continues to lead the way in the global development and exploitation of new areas of knowledge, utilizing the science-state concordat to maximize the delivery of economic, health and social benefits, on the one hand, and the advancement of scientific power on the other. In the case of genomic science, this task has been complicated by the need to combine its management of the changing governance of biomedical innovation with the epistemic integration of the new field of bioinformatics into its sphere of influence. Central to this process has been the propagation of modes of governance designed to ensure that, through bioinformatics, genomic science is able to order the process of knowledge production in a manner consistent with its interests.

**Figure 1.**
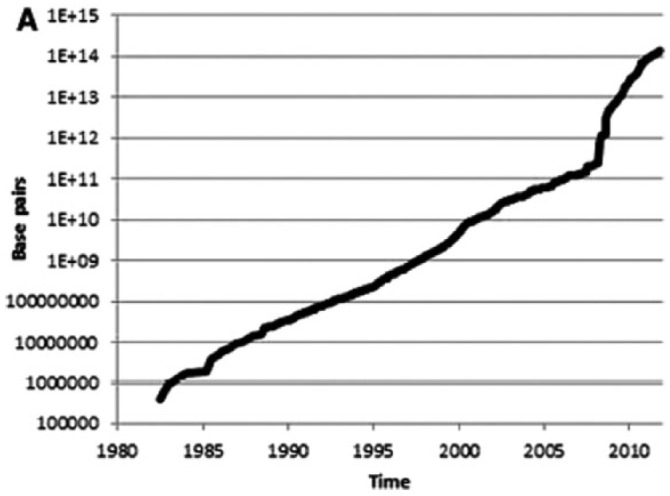
Cumulative base pairs in INSDC over time (Karsch-Mizrachi et al., 2012: Figure 2).

What was, of its nature, a challenging political task has been intensified by the sheer scale, complexity and exponential growth of the databases that form the core resource for the genomics project and the platform for the bioinformatics contribution. Important milestones in the evolution of a global network of databases can now seen to be the Protein databank (now the Worldwide Protein Databank or wwPDB) in the 1970s, the EMBL-Bank (DNA – now the European Nucleotide Archive) in 1980, GenBank (now part of US’s National Centre for Biotechnology Information [NCBI]) in 1982, and the DNA Databank of Japan (DDBJ) in 1986. From these separate initiatives emerged in 1987 the platform for a global infrastructure of database creation and storage through the formation of the International Nucleotide Sequence Database Consortium (INSDC), consisting of a partnership of EMBL-EBI, DDBJ and NCBI. The governance demands generated by this infrastructure are reflected in the rate of growth of the data it supports. Taking INSDC itself, Figure 1 shows the consistent exponential rate of increase of its data over time. Very similar rates of increase are evident in the latest database report from EBI (Cook et al., 2016: Figure 2).

**Figure 2.**
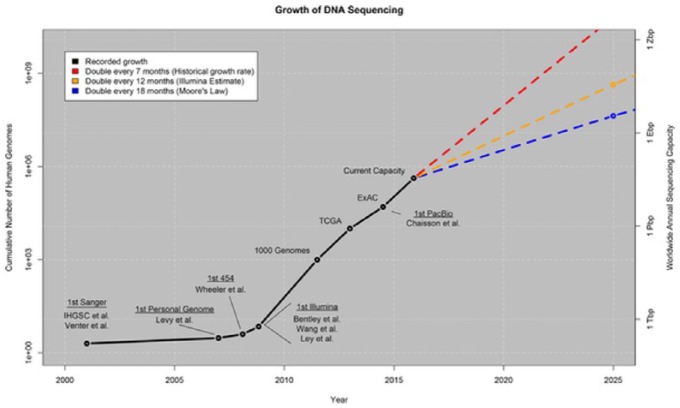
Growth of DNA sequencing (Stephens et al., 2015).

More generally, in the last decade the global quantity of stored sequence data has doubled approximately every seven months (Figure 2), with the result that biological data volumes will soon rival those produced by astronomical observation (Stephens et al., 2015; see also Kodama et al., 2012). In parallel, the engagement between the databases and their scientific users has also increased exponentially. EMBL’s (2015) figures show the one million requests per day in 2009 for EMBL-EBI services rising to eleven million in 2014.

The governance of this immense global enterprise had to be constructed from scratch. As Harvey and McMeekin (2008) observe, ‘From an institutional point of view, there was a void of norms on how the new streams of bio-data could come under public control, determining how such data could be produced, accessed, distributed and used’ (p. 39). Furthermore, the process of governance construction was as much a political as a technical challenge: to enable science simultaneously to keep control of the enterprise and exploit the potential of bioinformatics. Existing disciplinary interests had to be kept onside, and hence ‘[g]overning the HGP entailed not only constituting control relationships and material means for coordinating research, but also aligning the new regime closely enough with extant practices in molecular biology to make building this form of biology feasible’ (Hilgartner, 2013: 410). How did genomic science construct a system of governance for the definition and ownership of knowledge that maintained, enhanced and legitimated its power?

### Defining knowledge

For research funding markets to operate effectively, creating and trading knowledge, governance of the definition and ownership of a particular knowledge entity has to be agreed upon, disseminated and enforced. In bioinformatics, as in all science, the operation of both public and private economies of knowledge is dependent on an ontological infrastructure of agreed-upon measures, protocols, classificatory systems and technical benchmarks shared by laboratories to enable both collaboration and competition (Timmermans and Berg, 2003). Standards bind communities of practice together across space and across public and private sectors. Stable classificatory systems ensure that concepts and definitions are the same in every geographical location and cultural context, allowing the predictable communication and shared understanding and practice essential for market interaction (Timmermans et al., 1998). A leading UK bioinformatics manager summarized the governance issue thus:

Because from an industry perspective, the samplings for it just makes much more sense, when you’re working on a common standard. If you’re one of the players in the information supply chain, if you can provide the information to your customers in a format in a standard that they’re expecting, then that’s going to be an easier sell than if they then have to manipulate it in some way. But you get what are called these ecosystems of information. So you’ll have information that’s going from labs, to being analysed, to influencing something else. You get these ecosystems and it makes more sense if everybody is using the same standards. [Interview 31]

Another interviewee observed,

I think that definitely [standards creation] is a big rate-limiting step for innovation in this space because unless the standards are there, you can’t build the tools, and unless you have the tools you really can’t find out exactly what use cases are. So it’s a sort of chicken and egg problem. [Interview 9, bioinformatician]

The key political question is, who controls the creation of standards for the definition of the tradable knowledge in the operation of the bioinformatics market?

Reflecting on the role of publicly funded research in bioinformatics, Harvey and McMeekin (2008) argue that ‘the control over standards by the scientific community is a key feature of public appropriation’ and that ‘new institutions of control developed in response to new epistemic forms, the biodatabases’ (p. 66). How does such governance creation work where, as was the case in the early days of the new biodatabases, not only are the quality norms absent but the traditional paradigm of hypothesis-driven research is being replaced by the database-led approach of ‘discovery science’? The head of a UK bioinformatics institute provides an insight into the operation of self-regulation by science in this situation, describing those involved as constituting a ‘branch of science’ in their specialist work on ontologies and standards:

So people realized as soon as the technology to derive this data was there, that there would need to be standards and ontologies, and so a group of scientists around the world get together, they have a conference and say we need a standard, how are we going to do this? And then certain people come together and they form a committee, and they work, usually somebody will lead that and propose a standard and then it gets sent out to all of the people around the world, and they all comment, and eventually there is a paper that says: ‘This is the agreed standard!’ And that standard, in this case, was used to drive [database name], which is the database here, but there are various other databases in a consortium. It’s called the IMEX around the world that all use the same standards. So then once it’s agreed it becomes *de facto*, but it usually takes three to five years to do that. [Interview 22]

Emphasizing the significance of publicly funded science in this domain, the interviewee added: ‘There might be people in companies who get involved in that standards definition, but so far none of these standards have been driven primarily by companies; it’s been an academic exercise’.

If for ‘academic’ we read ‘public academic’ it becomes clear that the global, publicly funded bio-database institutions of the West and Japan, the NCBI, the EBI and the DDBJ, working through their collaborative platform, the INSDC, are driving what Harvey and McMeekin (2009) term the ‘political economy of self-regulation in bioinformatics’ through a ‘dominant, hegemonic presence’ (pp. 492, 502). From its inception, the INSDC has included governance creation as a key activity, drawing on the expertise of its constituent members to develop ‘core collaborative instruments’ (such as the Feature Table Document, the unified accessioning system, and the data model underlying the Sequence Read Archive) to organize and facilitate data definition, storage and access (Brunak et al., 2002; Karsch-Mizrachi et al., 2012). In parallel, the member organizations themselves continue to refine their own governance expertise; for example, the NCBI (2016) ‘develops and promotes standards for databases, data deposition and exchange, and biological nomenclature’.

A senior UK bioinformatician manager elaborated on how these global institutions build on their critical mass to maintain the momentum through further projects:

So big science projects like the ENCODE project, the 1000 Genomes Project, have really been taking bioinformatics forward … To take the 1000 Genomes Project, there was a whole set of new types of file formats that were developed and sorts of algorithms for doing various things as a result of the project… So you’ve got the funders, you’ve got the technology, the new types of next generating sequencing, the RNAseq as a key example of that, this is generating new algorithms, new software methods. [Interview 31, bioinformatician manager]

As fresh state-funded bio-databases have come on stream, they have been linked to the governance-development activities of these senior partners through a global network of scientific institutions tasked with harmonizing the ontologies of data in diverse databases. Organizations such as the Microarray Gene Expression Data Society, the Macromolecular Structure Database (part of the worldwide Protein DataBank), the Gene Ontology Consortium project and the Genomics Standards Consortium have defined the minimum information standards for any deposition process incorporating data into public domain databases. In the words of two of the scientists involved in their development, bio-ontologies exercise definitional power through the ‘formal representations of areas of knowledge in which the essential terms are combined with structuring rules that describe the relationship between the terms. Knowledge that is structured in a bio-ontology can then be linked to the molecular databases’ (quoted by Leonelli, 2012a: 51–52). Emerging in support of this mode of knowledge governance is the curator, tasked with creating bio-ontologies and adapting existing ones in order to organize data into a form capable of meeting the globalized research needs of bioinformaticians and biologists alike (Howe et al., 2008). With curators ‘well aware of the epistemic power of their classification systems’ (Leonelli and Ankeny, 2012: 32), governance control is thus exercised by science through the definition and propagation of shared constructs within which various communities in the ‘ecosystem of information supply chain’ [Interview 31, bioinformatician manager] agree to operate. In this manner, scientific knowledge production and governance norm production go hand in hand.

This is not to say that private industry does not have a role in the creation of standards governance in bioinformatics. It does, but it is obliged to work within the dominant agenda established by the public science projects and institutions. In their study of expressed sequence tag and single nucleotide polymorphism databases, Harvey and McMeekin (2008) note,

By establishing a global community of producers of data, constitutive of public production, the institution of an all-inclusive bio-database of a particular category gains a competitive advantage over partial databases. The more effective this process of inclusive bio-database growth, the less the scope for partial databases traded by commercial enterprises. (pp. 66–67)

From this economic advantage then flows the power to establish the measures of robustness and quality of data that govern the bioinformatics market (p. 67). What remains for the private sector is the servicing of the development of standards within the academic agenda. Hence an interviewee observed,

So I think that commercial companies do have an impact on standards, and actually not just the work that we’ve done, but if you look at the work of the Pistoia Alliance, a lot of that is focused on standards, and that’s a life sciences standards organization, which involves lots of commercial companies. [Interview 31, bioinformatician manager]

The hegemonic power of this mode of bio-knowledge standards governance, its ability to be both inclusive and exclusive in the operation of its rule systems, is apparent in the position of emerging economies, where the infrastructures in the life sciences are still developing. From a UK perspective, although the bioinformaticians interviewed would have frequent collaborations with scientists in the US, Europe and Japan, their collaboration with China and India is described in terms of potential and the provision of advice at best, rather than as regular interaction with equal partners. In large part, this is the result of the inability of Chinese and Indian scientists to achieve the technical standards required for access to international bio-databases. In the specific case of the databases of model organism biology, there is a much lower chance of incorporation of data from less prestigious, non-English speaking laboratories in developing countries and less chance of the scientists from such countries participating in the development of international databases (Leonelli, 2014: 6). As one might expect from the customary operation of hegemonies, the net effect of bioinformatics standards governance has been to render ‘genomics a selectively global industry, creating a specific map determined by Western science, technology, and government and economic interest’ (Thacker, 2006: 18).

### Owning knowledge

The governance of knowledge standards defines the parameters within which bioinformatics affects the process of genomic innovation, and knowledge ownership determines who benefits from the implementation of these definitions. The governance of what constitutes tradable knowledge in bioinformatics works happily within the accepted understandings, networks and institutions of the state-supported Western science hegemony adding fresh dimensions to this infrastructure as and when necessary. However, the governance of who owns the knowledge has proved much more contentious. Here in the domain of intellectual property, what Chow-White and Garcia-Sancho (2012: 144) term ‘the politics of the database’ has produced continuing institutional and ideological struggle with, on the one hand, scientists arguing that genomic information should be a public good husbanded through the stewardship of science and, on the other, entrepreneurs promoting the assumptions of proprietary business models where private ownership is a necessary condition of workable markets.

At a meeting in Bermuda in February 1996, attended by the Wellcome Trust, the NIH National Centre for Genome Research, the US Department of Energy, the Human Genome Project of Japan, the German Human Genome Project, the UK Medical Research Council and the European Commission, new rules were set out for the deposition of genomic data as a precondition for international collaboration between contributing laboratories to the human genome project (Bentley, 1996). Private industry was not present. The agreed policy stated that

all human genomic sequence information, generated by centres funded for large-scale human sequencing, should be freely available and in the public domain in order to encourage research and development and to maximise its benefit to society. (Human Genome Organisation, 1996: 1)

What was important, the policy made clear, was ‘to prevent such centres establishing a privileged position in the exploitation and control of human sequence information’ (Human Genome Organisation, 1996: 1) – that is, privately owning and exploiting the data. Supported by the powerful alliance of state and scientific actors behind its initiation, the policy was then propelled across the global research funding market and established as the dominant form of governance for genomic information, thus reshaping scientific practice (Kyrpides, 2009). Kaye sums ups its effect as creating a climate ‘in which data sharing has become the default and [grant] applicants must demonstrate why their data should be exempt from the requirement that it should be deposited for use by other scientists’ (Kaye et al., 2009: 332).

The political ambition of the policy was as much ideological as it was technical, designed to establish and maintain the governance power of publicly funded genomic science through the exploitation of particular vocabularies of justification in the lexicon of scientific values. It builds in particular on the ideological theme of ‘communism’, in the sense of common ownership, which Merton (1973) describes as an ‘integral element of the scientific ethos’: ‘The substantive findings of science are a product of social collaboration and are assigned to the community. They constitute a common heritage in which the equity of the individual producer is severely limited’ (p. 273). In his critique, Mulkay (1976b) argues that such norms in fact constitute the delineation of part of an occupational ideology.

In the case of bio-data, science’s promotion of ‘communality’ as the value that should guide the governance of genomics and bioinformatics sought to keep the control of the new knowledge within the domain of publicly funded science. This approach immediately collided with an established and state-sponsored approach to the ownership of knowledge. Prior to the adoption of the Bermuda Principles, the data release policies of most government-funded projects in the US and UK allowed researchers to retain their data privately until publication of results or for some specified ‘exclusivity period’, usually about one year (Contreras, 2011: 66). Regardless of the preferences of scientists, in an early manifestation of the subsequent ‘translation’ debate, governments had begun to insist that the requirements of commercialization, including the use of intellectual property rights, should form an integral part of university research activity (Powell and Owen-Smith, 1998). The 1980 Bayh-Dole Act in the US, for example, exercised a profound effect on university researchers by explicitly encouraging them to seek formal property rights over their knowledge outputs through patents (Mowery and Ziedonis, 2002). Meanwhile in the sphere of genomics itself, the controversy over what genomic information could, or could not, be patented had been a prominent feature of the governance debate from the early 1980s onwards (Eisenberg, 2002).

The attempt by genomic science and bioinformatics to reclaim the governance territory of knowledge ownership and place it securely within their joint domain brought science’s view of bio-data as a public good into sharp collision with the industry view that private ownership of genomic knowledge was an essential condition of progress in the field. Perhaps the most celebrated example of this continuing tension was the technological ‘arms race’ and political struggle between the HGP and Craig Venter’s Celera Genomics to be the first to sequence the human genome (Shreeve, 2004). More prosaic, but more important in the long run, was the ability of scientific actors to draw on their access to state governance institutions to shape the governance agenda. In the early 1990s, the initial patent-friendly attitude of the NIH towards the seeking of patents on expressed sequence tags provoked what Cook-Deegan (1994) has described as ‘an international firestorm’ of protests from international science (pp. 330–331). Suitably impressed, the NIH responded by transiting rapidly to a position where it was able actively to support the creation of the anti-patenting Bermuda principles in 1996. This shift is reflected in the experience of the National Human Genome Research Institute (NHGRI - the principal US funding agency for genome research and part of NIH). Contreras (2011) describes how ‘in the negotiations at and leading up to the Bermuda meeting, the scientific community’s acknowledgement of the collective norms of data sharing and the public domain, bolstered by the gravitas of several Nobel laureates and other leading figures, seems to have captured the agency’s imagination’ (p. 88). Thereafter, the view that genomic data should be treated as a public good widely available and unencumbered became ‘ingrained as part of NHGRI’s basic position’, effectively overriding its statutory obligations under the Bayh-Dole Act (Contreras, 2011: 88; see also Rai and Eisenberg, 2003). The US science-state concordat had done its work and proved its political value to science through the promotion of an ideological position supportive of a particular scientific elite. In contrast, the values of commercialization, ownership and private profit had suffered a serious rebuff.

The rebuff was strengthened by the ability of the traditional scientific value of communality to ally itself with other ideological themes in the political hinterland of bioinformatics. Thus ‘bio-data as a public good’ resonated easily with a powerful cocktail of emerging themes in the bioinformatics discourse such as democratizing the data (Chow-White and Garcia-Sancho, 2012: 148–152), public participation in genetic databases (Lassen and Jamison, 2006; Tutton, 2007), open access (Holdsworth, 1999), opposing the ‘power geometry’ of the uneven global distribution of knowledge resources (Thacker, 2006) and hackers as heroes (Marturano, 2003). At the level of international institutions, patenting of the human genome faced continuing ideological opposition. In 1997 the *Universal declaration on the human genome and human rights* by the International Bioethics Committee (IBC) of UNESCO initiated a global debate about the moral statuses of the human body and human life and their relationship to the market; this debate has continuing momentum in bioethical and policy-making circles today. At the conclusion of its 8th Session in 2001, the IBC adopted by consensus an *Advice on the patentability of the human genome*, which states that ‘there are strong ethical grounds for excluding the human genome from patentability’ and further recommends

that the WTO, in its review of the TRIPS Agreement, clarify that, in accordance with the provision of Article 27(2) (the morality clause), the human genome is not patentable on the basis of the public interest considerations set out therein, in particular, *ordre public*, morality and the protection of human life and health. (IBC, 2002: 1)

Ethical discussions regarding the status of DNA, the human embryo, human dignity and the commercialization of the human body, often subsequently enshrined in national legislation and, in the case of the Council of Europe’s *Convention on human rights and biomedicine*, in a protective international agreement now form an internationally salient discourse with which patenting policy and practice in bioinformatics is obliged to engage (Salter and Salter, 2013). At the same time, other international developments, in particular the *Convention on biological diversity*, have emphasized the importance of the communal ownership of knowledge as a counterbalance to what some have termed the ‘biopiracy’ of the developing world’s knowledge by Western countries (Robinson, 2010: Chapter 2).

Governance compromises regarding the degree and timing of public knowledge ownership were nonetheless made as the quantity, geographical spread and complexity of bioinformatics infrastructure increased. For example, in its 2008 policy on *Data release and resource sharing*, Genome Canada (2008) ‘recognizes publication as a vehicle for data release’ (the right to protect the interest of the individual scientist) and ‘the need to protect patentable and other proprietary data’ (p. 1). Similarly, the NHGRI Encyclopedia of DNA Elements (ENCODE) project policy offers some protection for the data generators through the recommendation of a nine month embargo period during which users of released data are requested not to publish or present results based on that data (ENCODE, 2009: 4). There is not ideological harmony within the scientific community, where individual self-interest may conflict with the dominant view of ownership. A senior GlaxoSmithKline bioinformatics manager involved in joint research with UK university scientists into Alzheimer’s, having advocated the view of bio-data as a public good, then commented,

We did get quite a bit of pushback from some of our academic institutes; they felt that there might be intellectual property there that could be obtained and some of their researchers may make that finding and may identify double biomarkers, and identify subgroups of patients with Parkinson’s disease, so they were keen to be able to generate intellectual property on that. So we ended up sort of compromising. [Interview 32]

## Governance control and professional rivalry

Whilst the science-state relationship may be able to resolve such tensions in the governance of knowledge ownership, other tensions adjacent to that relationship are less amenable to compromise. Commenting on the factors necessary for the successful translation of genomic information into patient benefit, the head of a patient organization in the genomics field observed,

So you need the biological research, you need the bioinformatics, you need the infrastructure and you need the workforce and, fifthly, *you need the understanding of patients and public of the legitimacy of this approach, so there’s a public communication job here as well.* [Interview 1 – original emphasis]

Awareness is growing of the complexities of governance as a part of scientific work, where scientific elites have to negotiate directly with publics in order to achieve the legitimacy science needs (Wellcome Trust, 2009). Reflecting on the linking of genomic and primary care data in the UK, the head of a bioinformatics department noted,

And there are many, many regulations that need to be put in place about the ethics, about privacy, about how to share this data, about how to store this data, who will store this data … what’s the legislation behind it. There is a big gap around all of this, so at the moment there’s a lot of science, there is a lot of technology, there is a lot of intelligence, there are many methodologies and we are in a very good position, especially in England, to really take advantage of all of this soon. But there is a complete lack of legal and regulatory governance, political, ethical, social infrastructure to kind of make that happen at the moment. [Interview 34, head of bioinformatics department]

The difficulty faced by genomics and bioinformatics is that for governance structures to be developed, there must be some translation of results into health benefits, resulting in public trust; in addition, genomic and clinical data have to be integrated and knowledge ownership agreed upon across the separate and distinct governance territories of scientists, clinicians and patients. As the director of a bioinformatics institute noted, ‘Suffice it to say that the interface between basic biology knowledge and data, and the clinical world is a difficult one to bridge, and that’s where we’re at at the moment’ [Interview 22]. In negotiating this complex political route, moving beyond the comfort of its private *in silico* zone where self-regulation is the norm and interacting directly with the messy preferences of the wider public, science is to an extent constrained by the assumptions of its core ideology.

Reflecting on the scientist-clinician divide in the field of stem cell translational research, Cribb et al. (2007) note that ‘the normative structures produced by the institutions and organisations of the scientific and the clinical construct different ethical spaces and role positions’ reinforced by the institutional accountabilities to their respective, and quite distinct, professional communities (p. 353). Scientists are accountable to the peer review mechanisms and hierarchies of science, clinicians to the regulatory bodies of medical practice. Their capacity to work harmoniously together, construct new forms of governance and negotiate the resolution of differences in their common pursuit of the benefits of bio-data integration is therefore limited by their distinct identities, governance territories and professional responsibilities (Wainwright et al., 2006).

In addition, clinical medicine’s internal culture and epistemic rivalries are at least as dense as those of science, generating added complexities in the gestation of new governance. In his study of the development of standardized guidelines in the French oncology sector, Castel has documented how competition between rival groups of physicians intent on improving their relative positions or ‘jurisdictions’ at the expense of others served to structure the politics of how the guidelines were produced. Standards were used as ‘strategic resources’ in the pursuit of political gain (Castel, 2009; see also Abbott, 1988; Timmermans and Berg, 2003). Similarly, in their work on data monitoring committees, Keating and Cambrosio (2009) show how the production of governance relied on institutions to facilitate ‘the formation of an internal consensus as to how to proceed “objectively” rather than on the production of abstract standards, norms and measures that express the regulatory ideal of objectivity’ (p. 326).

It is doubtful if all the scientists now engaging with clinicians in the bringing together of genomic and patient data are fully aware of the political nuances of governance creation in a medical domain where power trading is endemic. The head of a UK bioinformatics institute, comparing the openness of data in basic genomics science with the restricted accessibility of clinical data in the UK National Health Service (NHS), illustrated the nature of the cultural differences, saying,

But in the clinical world this [open access to data] is not true. Clinical data is totally controlled by the clinicians, and this is going to change. It will change. They say, ‘Oh, it’s people’s data, we can’t let this people’s data out!’ Most people now feel that this is a smokescreen and people are just protecting their own thing. These data have been collected by public money and should be made available for the public good, I think. [Interview 22]

Such over-optimism, or perhaps professional hubris, is balanced by an awareness among other scientists of the need for caution. One observed,

So we need to rethink architectures in terms of how genomic data is stored. I think we should go with the assumption that we have to protect it like health information, but for research it’s not going to work so how do you de-identify it enough so that research doesn’t suffer? [Interview 9, bioinformatician]

In the UK, the ramifications of not being cautious and instead happily applying the traditional scientific view of data ownership to the complex and sensitive world of patient data in the NHS are manifest in the furore surrounding the attempted introduction in 2014 of the Care.data programme, an initiative to combine the primary care and hospital personal medical records of NHS patients within the national Health and Social Care Information Centre database. Within weeks of its launch, public and professional concern was so overwhelming that the implementation of the scheme was delayed for six months and is now going through an extensive process of consultation and piloting (Taylor, 2014; Triggle, 2014). In response to longstanding pressure from the medical research community for the better use of patient records as a research resource, in particular that generated by the Academy of Medical Sciences’s (2006) report *Personal data for public good: Using health information in medical research*, one of the programme’s main policy objectives was: ‘To drive economic growth by making England the default location for world-class health services research’ (Carter et al., 2015: 405). As a result of the comprehensive opposition to the programme, that objective has now been removed and replaced with ‘To better understand diseases and develop drugs and treatments that can change lives’ (National Health Service England, 2015), clearly a less science-oriented and more patient-friendly framing.

There is what may be termed a ‘legitimation gap’ presently evidenced in the difficulties associated with the construction of large scale databases geared to the needs of science as well as to the needs of patient care, situated within the cultural territory of the clinician (Carter et al., 2015). Researchers have tended to assume that the public benefits of science are obvious to the public and that trust will naturally follow. Up to a point, such cultural naivety is to be expected, genomics and bioinformatics have little experience of adjusting their customary approach to the governance of knowledge ownership to the highly politicized context of the NHS and the internecine niceties of clinical medicine’s own approach to cultural power. What is then clear is that genomic science has much knowledge governance work to do and many negotiations to resolve if societal trust is to be retained and science’s control of knowledge production assured.

## Conclusions

In order to improve their fields’ chances of success in the research funding market, scientists are accustomed to making promises where the delivery or timescale are problematic. Thus a common political problem for scientific domains becomes one of maintaining the legitimacy of the field when expectations are not met. In this situation, the re-engineering of those expectations becomes an integral part of the generation of new knowledge and knowledge governance becomes an essential political tool in enabling such re-engineering to occur. In the case of genomic science in the late 1990s, bioinformatics presented itself as the natural solution to the expanding legitimation gap between the promised world of genomic medicine and the reality of the exponential increase in the deluge of bio-data.

However, the recruitment of the new field of bioinformatics as political handmaiden to the ambition of genomic science, in concert with the maintenance of control, was not straightforward. It was possible that private industry would seize the opportunity and reap the benefits of the translation of genomic data into new forms of clinical medicine. To deal with this threat and integrate bioinformatics on its preferred terms, genomic science established its preferred governance structures for knowledge definition and ownership, which were embedded in its system of self-regulation. The objective was to keep control of the genomics enterprise within the publicly funded domain of science. In implementing this strategy, genomics had three main advantages. First, its Western science base was dominant, secure and unlikely to be challenged by alternative governance proposals from the much weaker science-state partnerships of the emerging economies with potentially different interests. Second, its transnational networks of scientific institutions proved themselves adroit from very early on at evolving new forms of knowledge governance capable of addressing the definitional and ownership issues associated with the new bio-databases in ways protective of the scientific interest. Third, the ideological resonance between the bioinformatics trope of ‘bio-data as a public good’, the traditional scientific value of communality and a range of anti-private ownership values (e.g. open access, democratizing the data, special status of the human genome) proved a powerful legitimation tool to set against the ambitions of private industry.

Within the comfort zone of the science-state concordat, genomics and bioinformatics have found the construction of the required modes of new knowledge governance relatively straightforward. Where the difficulties have arisen has been in genomics’ engagement with the domain of the clinician in pursuit of the integration of genomic and patient data without which the promised world of genomic medicine cannot be created. Practice-based clinical medicine has its own distinct professional identity, institutions, values and interests to protect and, if there are resources available, advance. Unlike genomic science, its ideology and practice means that its engagement with society, and society’s preferences, is close and routine rather than distant and promissory. Society, meanwhile, is drawn into the equation by genomic science’s need for individualized patient data. As the political mix becomes more complex so the ability of bioinformatics to maintain the genomics momentum whilst keeping control of the field becomes more dependent on the construction of new modes of knowledge governance. The global dimension may be important here. Although generally accepting of Western leadership in genomic science, countries such as China and India have shown themselves quite prepared to pursue their own practice-based approach to biomedical innovation in other fields, such as regenerative medicine, in defiance of the preferences of Western science (Salter et al., 2016). It may be that they would sense a similar opportunity in genomics. Thus as the political mix becomes more complex and more global, it remains to be seen whether genomic science has the ideological muscle, governance expertise and political skill to legitimize and implement the next phase in its promissory journey.
